# Nanocarriers for delivery of siRNA as gene silencing mediator

**DOI:** 10.17179/excli2022-4975

**Published:** 2022-08-01

**Authors:** Aideé Morales-Becerril, Liliana Aranda-Lara, Keila Isaac-Olivé, Blanca E. Ocampo-García, Enrique Morales-Ávila

**Affiliations:** 1 Laboratorio de Toxicología y Farmacia, Facultad de Química, Universidad Autónoma del Estado de México, Toluca, Estado de México 50120, México; 2 Laboratorio de Investigación en Teranóstica, Facultad de Medicina, Universidad Autónoma del Estado de México, Toluca, Estado de México 50180, México; 3 Laboratorio Nacional de Investigación y Desarrollo de Radiofarmacos-CONACyT, Instituto Nacional de Investigaciones Nucleares, Ocoyoacac, Estado de México 52750, México

**Keywords:** siRNA, nanocarrier, drug delivery systems, nanomedicine

## Abstract

The term nanocarrier refers to sub-micrometric particles of less than 100 nm, designed to transport, distribute, and release nanotechnology-based drug delivery systems. siRNA therapy is a novel strategy that has great utility for a variety of treatments, however naked siRNA delivery has not been an effective strategy, resulting in the necessary use of nanocarriers for delivery. This review aims to highlight the versatility of carriers based on smart drug delivery systems. The nanocarriers based on nanoparticles as siRNA DDS have provided a set of very attractive advantages related to improved physicochemical properties, such as high surface-to-volume ratio, versatility to package siRNA, provide a dual function to both protect extracellular barriers that lead to elimination and overcome intracellular barriers limiting cytosolic delivery, and possible chemical modifications on the nanoparticle surface to improve stability and targeting. Lipid and polymeric nanocarriers have proven to be stable, biocompatible, and effective *in vitro*, further exploration of the development of new nanocarriers is needed to obtain safe and biocompatible tools for effective therapy.

## Introduction

The term nanocarrier refers to sub-micrometric particles less than 100 nm, designed to transport, distribute, and release molecules with biological activity. These drug delivery systems are based on nanotechnology and are identified as promising strategies used to overcome technical, biological and biopharmaceutical limitations, having among their advantages, the possibility of designing multifunctional drugs with high therapeutic efficacy, thanks to the possibility of increasing specificity and selectivity for cellular or molecular targets.

siRNA therapy is a novel strategy that has great utility in some chronic diseases, however, it has been observed that the delivery of naked siRNA has involved great difficulties, due to some of its physicochemical properties and its repercussions on biological behavior, such as its rapid degradation in biological fluids, its non-specific accumulation in tissues following systemic administration, its inability to penetrate cells by passive diffusion, and its short half-life of less than five minutes in plasma due to its susceptibility to nucleases (Sajid et al., 2020[128]; Cullis and Hope, 2017[32]). 

**Figure 1:**
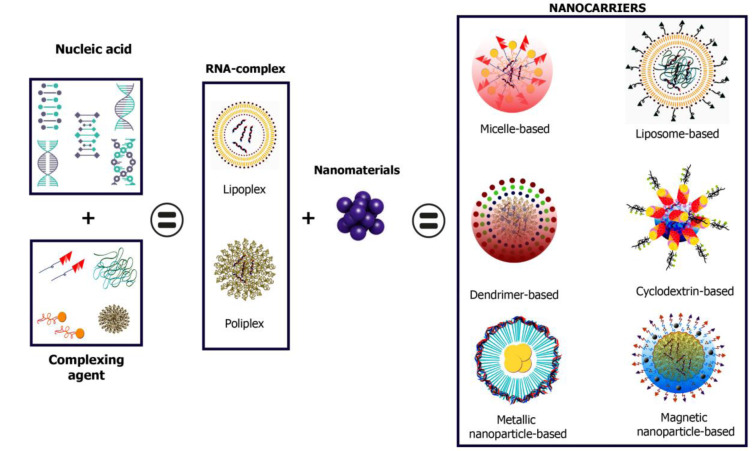
siRNA delivery systems can be constructed from a variety of materials with varying physicochemical features and biological behavior.

The most prominent candidates for siRNA delivery are nanoparticle (NP) systems. siRNA can be incorporated into an NP formulation through covalent bonds with NP components or by electrostatic interactions with the NP surface, as acids in strongly negatively charged nuclei tend to form complexes. In addition, NP has been considered as specific and safe nanocarriers since they offer a set of advantages such as a high surface-to-volume ratio, a significant increase in bioavailability and a decrease in clearance of low bioavailable active ingredients (APIs), as well as their ability to preferentially accumulate on a selected target (see Figure 1(Fig. 1)) (Mainini and Eccles, 2020[96]).

Nanocarriers based on nanoparticle formulations allow organ-specific targeting and provide a wide versatility to package siRNA with multifunctional performance due to their surface modifications, thus enabling the delivery of macromolecules via cellular and even transcellular pathways. In addition, it is suggested that nanoparticle systems can promote endosomal escape by different pathways such as the “proton sponge effect”, membrane fusion, membrane destabilization or induced swelling, thus preventing late endosome elimination in conjunction with API, which is very useful for siRNA delivery, since it enters cells by endocytosis like most nanoparticles (Lin et al., 2020[88]; Ashrafizadeh et al., 2020[10]; Kim et al., 2019[73]; Chevalier, 2019[30]; Smith et al., 2019[140]; Singh et al., 2018[139]).

Interest in the development of siRNA nanocarriers began with gene therapy through the transfer of nucleic acids, including siRNA, microRNA (miRNA), short hairpin RNA (shRNA), antisense oligonucleotides (ASOs), aptamers, mRNA, plasmid DNA (pDNA), and CRISPR-Cas9. Exponential growth in the areas of molecular biology, pharmaceutical technology and materials science has enabled the design of effective pharmaceutical formulations that are currently in clinical trials and commercialization.

siRNA delivery systems can be constructed from a variety of materials with varying characteristics (Figure 1(Fig. 1)), all of which contribute to maximizing therapeutic potential. For this review, we will present and classify only systems composed of lipidic, polymeric, and inorganic nanoparticles, which make up micelles, liposomes, polymer blocks, hydrogels, etc., each with different physicochemical properties that allow for specific siRNA charge depending on the type of nanoparticle (Sharma et al., 2020[135]; Chenthamara et al., 2019[28]).

Small interfering RNA (siRNA) is a non-coding RNA-type oligonucleotide (ncRNA; ~2 nm and ~13.5 kDa), its role in mediating post-transcriptional gene silencing has been widely studied and it has been established that binding to the RNA-Induced Silencing Complex (RISC, multi-protein complex) guides the specific degradation of messenger RNA (mRNA) preventing its translation into a protein. There are six siRNA drugs in late stages of Phase 3 clinical trials, including *vutrisiran, nedosiran, fitusiran, teprasiran, cosdosiran*, and *tivanisiran*. The use of siRNA in recent decades has become a promising therapeutic alternative to address gene overexpression for various pathological conditions, providing significant advantages regarding pharmacological inhibitors, highlighting its specific binding activity; meaning that siRNA can selectively bind to a target mRNA allowing the silencing of desired genes (Kokkinos et al., 2020[80]; van den Brand et al., 2018[153]; Sarkies and Miska, 2014[131]; Lin et al., 2020[88]).

In summary, there are 16 approved nucleic acid drugs: 9 ASO-based, 4 siRNA-based, 1 aptamer-based, and 2 mRNA-based (the latter being *Tozinameran* developed by Pfizer/BioNTech and *Elasomeran* developed by Moderna, which were designed for the prevention of coronavirus-19 (COVID19), they were approved at the same time in 2020) (Paunovska et al., 2022[116]; Zhuang and Cui 2021[178]; Hodgson, 2021[57]; Ferenchak et al., 2021[38]).

## Nanocarriers Composed of Lipid Nanoparticles

Lipids have the natural tendency to enhance cellular uptake of siRNA, with the added advantage of being very simple to formulate, have great versatility in their function, with diverse and programmable release profiles, just by modifying their lipid matrix and functionalizing molecules. Lipid nanocarriers are generally biodegradable, biocompatible, non-immunogenic or low immunogenic and have tolerable or low toxicity. However, in some cases, these nanocarriers are not completely inert, because some cationic lipids (amphiphiles with quaternary ammonium head groups) can reduce mitosis in cells, form vacuoles in the cytoplasm, and cause detrimental effects on key cellular proteins such as protein kinase C. On the other hand, notable disadvantages include their limited stability, their relatively low capacity to load siRNA, and, occasionally, the possible interaction and breakdown of payloaded nucleic acids (Han et al., 2021[51]; Inglut et al., 2020[61]; Scheideler et al., 2020[133]; Zatsepin et al., 2016[173]; Tenchov et al., 2021[148]).

**Figure 2:**
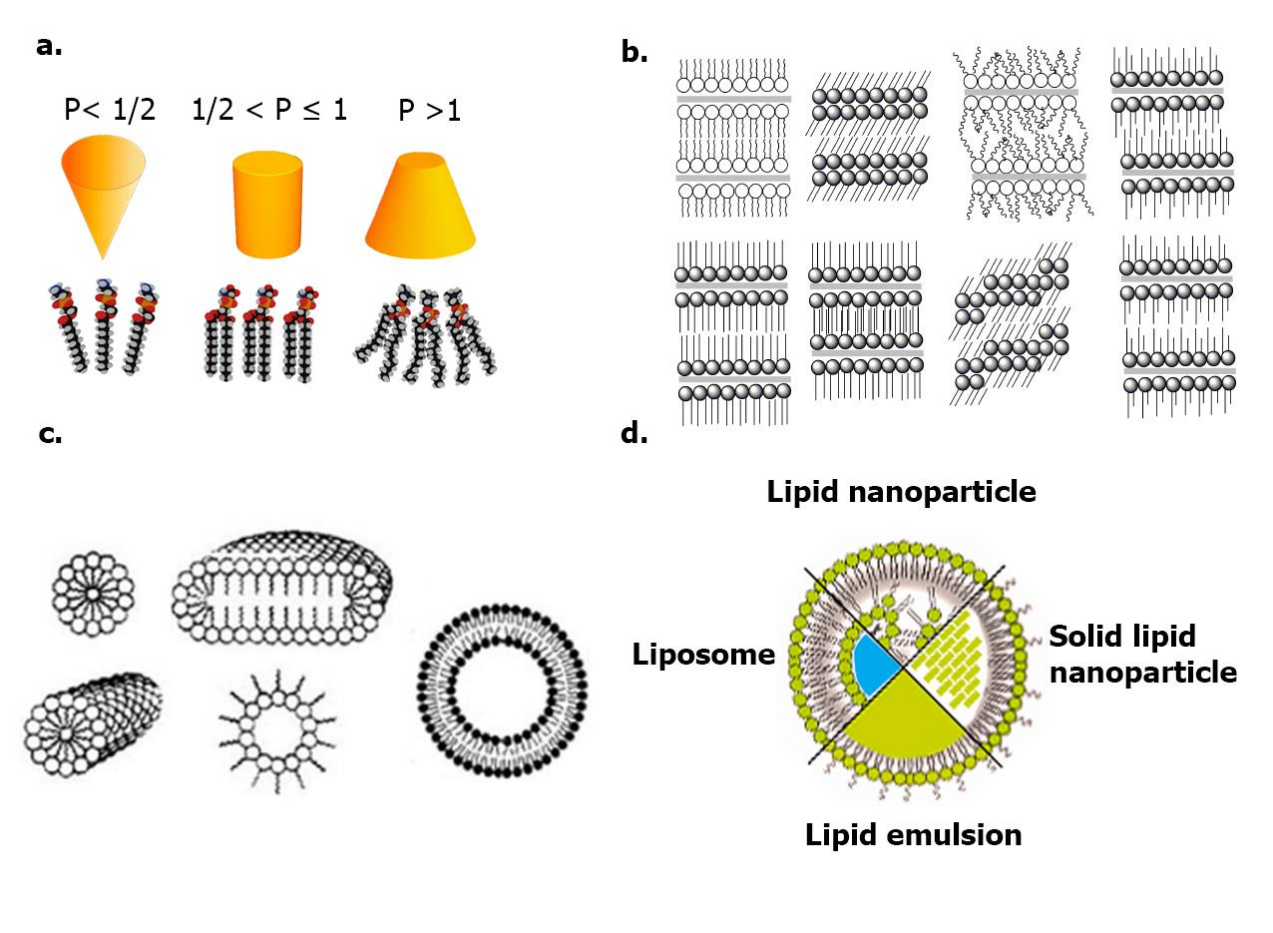
a. Lipid packing parameters and phases (micellar, bilayer, hexagonal); b. Varieties of lipid phases (lamellar, sub-gel, gel, liquid crystalline, etc.); c. Lipid self-assembly aggregates (Koynova and Caffrey, 1998); d. Lipid nanocarriers for delivery

RNA lipid nanocarriers are called lipoplex, which refers to systems composed of a combination of cationic lipids with nucleic acids, their formation consists of two steps, first, the cationic lipidic environment promotes electrostatic interactions, while the second step concerns the rearrangement and condensation of the lipoplex, forming structured self-assembly between lipids and phosphate groups from the siRNA main chain. The geometry of lipids determines the phase structure (micellar, lamellar, cubic, and inverted hexagonal phase) according to the packing parameter, moreover, it is well known that the phase plays a significant role in physicochemical and biological behavior, determines digestibility, absorption pathway, distribution, uptake, and delivery mechanisms (see Figure 2(Fig. 2); Reference in Figure 2: Koynova and Caffrey, 1998[81]). The most common lipid nanocarriers for siRNA delivery are micelles, liposomes, and lipid solids, but other lipid formulations are currently being studied and will later be discussed (Berger et al., 2021[15]; Fairman et al., 2021[37]; Kokkinos et al., 2020[80]).

### Micelles

Micelles are amphiphilic systems of small lipid vesicles with spherical shape, they have a hydrophobic core and a hydrophilic shell, they are produced by spontaneous self-assembly in aqueous media, their formation depends on amphiphile concentration, temperature, solvent, and size of hydrophobic/ hydrophilic domains, they can protect RNA/DNA and/or drugs in their micellar core, due to their small size (≤ 100 nm), they are applied for siRNA release, in most cases, they are conjugated with polymers to avoid binding to negatively charged serum proteins, also to prevent their aggregation, and to provide steric stabilization (Ojo et al., 2021[109]).

### Liposomes

Liposomes are large, closed spherical vesicles constructed from a lipid bilayer, which could be classified according to their size (~0.025-5 μm) and their number of lipid bilayers (unilamelar or multilamellar vesicles), composed of different types of phospholipids, cholesterol and steroids bounding the hydrophilic core. Being formed by the self-assembly of amphiphilic molecules, the components are arranged so that they can be used as nanocarriers for both hydrophobic and hydrophilic components. These systems have a high degree of biocompatibility, degradability, efficacy, encapsulation capacity for plenty of APIs and ease of formulation. Liposomes serve as smart release systems in conjunction with various functionalizing agents, so they have been the standard for siRNA transfection (Ajeeshkumar et al., 2021[4]; Aldosari et al., 2021[5]; Majumder and Minko, 2021[97]; Charbe et al., 2020[24]; Bholakant et al., 2020[16]).

### Solid lipids nanoparticles

Other lipid nanocarriers are those composed of solid lipid nanoparticles (SLNs) with a size of around 100-200 nm, which are micellar vesicles formed by colloidal nanoparticles grouped in a lipid monolayer enclosing a solid, hydrophobic lipid core; they are formed after emulsion with a surfactant that stabilizes the lipid dispersion, their function is to prevent permeation and degradation of their components, they have the advantage of being highly biocompatible, moreover, they have good storage stability and provide the opportunity to carry out a sterilization and lyophilizing process if required, therefore, this type of nanocarrier can incorporate lipophilic or hydrophilic molecules such as siRNA following several strategies (Basha et al., 2021[13]; Dhiman et al., 2021[34]; Khalid et al., 2020[70]; Yonezawa et al., 2020[170]; Scheideler et al., 2020[133]).

### Miscellaneous lipid nanoparticles for siRNA delivery

The most commonly used lipid nanocarriers are liposomes, solid lipid NPs and nanostructured lipid carriers, among others, all of which have long-term physicochemical stability as nano-emulsions. Table 1(Tab. 1) (References in Table 1: Ahmed et al., 2021[3]; Gao et al., 2021[43]; Habib et al., 2021[50]; Hattori et al., 2020[53]; He et al., 2015[55]; Jyotsana et al., 2019[66]; Lu et al., 2019[93]; Qu et al., 2014[123]; Sánchez-Arribas et al., 2020[130]; Shi et al., 2017[136]; Shin et al., 2020[137]; Xu et al., 2013[163]; Yu et al., 2012[171]) summarizes the reported lipid nanocarriers of nucleic acids. The droplet size range is 55 to 209 nm, with toxicity of less than 30 %, and *in vitro *gene knockdown ranging from 50 to 98 %. These nanocarriers are mainly designed for breast cancer therapy, and the predominant routes of administration are parenteral (intravenous (IV) or intratumoral (ITI) injection). Hybrid systems have been reported, composed with other types of nanoparticles such as polymeric ones, mainly forming liposomes that promote structural modifications with PEG to increase stability in plasma and avoid non-selective adhesion or, similarly, lipid nanocarriers with surface modifications with peptides, proteins, antibodies or aptamers (Herceptin or hyaluronic acid) that act as ligands to direct the nanocarriers to specific targets are observed (Rehman et al., 2020[124]; Scheideler et al., 2020[133]). The nanocarrier proposed by Ball et al. (2018[12]) provides an example of a lipid nanocarrier designed for oral administration of siRNA, composed of a mixture of lipoid 3060, cholesterol, DSPC, and PEG 200-DMG with a size of about 140 nm, PDI of 0.12, and a ζ potential of ± 10 mV. Unfortunately, this nanocarrier did not effectively knockdown GAPDH *in vitro *and *in vivo* in Caco-2 cells and in the mouse model respectively, demonstrating that gene silencing efficiency may be affected mainly by pepsin, bile salts, and mucin concentrations, when the nanocarriers are administered orally since the nanocarriers are destabilized, altered, and trapped in the gastrointestinal (GI) tract environment.

## Polymer Nanoparticle-Based Nanocarriers

Polymer-based nanocarriers represent the second most widely used carrier type for siRNA delivery and are more robust and stable nanocarriers than lipid nanocarriers, generally referred to as polyplex, which is a complex between the cationic groups of the polymer and the phosphate group of the nucleic acid. Polymeric nanoparticles can be used to protect siRNA by modifying its ionizable groups or by varying its size, consequently, siRNA increases its absorption rate and they can be classified as vesicular systems (nanocapsules) and matrix systems (nanospheres) (Castro et al., 2022[22]; Kim et al., 2021[78]). In the particular case of siRNA, polymeric systems condense or form a complex with siRNA, as a consequence, these nanocarriers can be found in the form of micelles, polymersomes, dendrimers or cyclodextrin polymers (Witika et al., 2020[160]; Vasile, 2019[154]; Castro and Kumar, 2013[21]).

These nanocarriers are mainly composed of cationic or ionizable polymers (see Table 2(Tab. 2); References in Table 2: Chen et al., 2015[26]; Gallon et al., 2015[41]; Kim et al., 2009[77], 2013[74]; Li et al., 2015[87]; Noh et al., 2011[107]; Pangburn et al., 2012[114]; Qiu et al., 2019[122]; Wang et al., 2018[157]; Zheng et al., 2019[175]; Zou et al., 2017[181]), to protect the siRNA payload and increase its cellular uptake, thus finding systems with linear, branched and/or block copolymers that have the ability to bind siRNA through covalent bonds (linear and branched copolymers) or bind through their amphiphilic properties to encapsulate siRNA (block copolymers) (Patel et al., 2021[115]; Ahmed et al., 2021[2]; Itani and Al Faraj, 2019[63]). 

### Polymeric micelles

Polymeric micelles are supramolecular self-assemblies with different morphologies (spheres, discs, and worm-shaped assemblies), composed of amphiphilic synthetic macromolecules in which the individual block copolymers are generally linked by non-covalent interactions, solubilizing the API in their core, while their shell allows them to be suspended in the aqueous medium. Polymeric micelles are considered a good system siRNA delivery because they use the core-shell structure for delivery and are smaller in size (< 200 nm) and more efficient for cellular internalization than other polymeric nanocarriers, they also have a high loading efficiency, are versatile, stable under physiological conditions and can be divided into two categories: (1) micelles formed from direct binding of polymers via covalent (non-degradable) bonds to siRNA and (2) micelles formed from direct condensation of siRNA with amphiphilic polymer block (Wan et al., 2021[155]; Ghezzi et al., 2021[46]; Charbe et al., 2020[24]).

### Polymersomes

Polymersomes are characterized as spherical cavitary bodies with a bilayer membrane between 2-47 nm in size, morphologically similar to lipid-based vesicles but consisting of amphiphilic block copolymers. These nanocarriers show a lower permeability to water and can tolerate much more areal pressure before rupture. Consequently, they are resistant, stable and are used to administer both hydrophilic and hydrophobic APIs, how-ever, their slow release may sometimes be a disadvantage due to their membrane thickness. On the other hand, thanks to the characteristic self-assembly of amphiphilic block copolymers, they can maintain their well-defined structure in an aqueous media promoted by a thermodynamic phenomenon between non-covalent physical interactions. Due to the physicochemical versatility of polymersomes, their increased stability and improved payload retention, they are used for the delivery of nucleic acids and/or macromolecules for both *in vitro* and *in vivo* delivery (pDNA, AON, siRNA) (Scheerstra et al., 2022[132]; Araste et al., 2021[8]; Moulahoum et al., 2021[104]; Iqbal et al., 2020[62]).

### Dendrimers

Dendrimers are a class of highly stable spherical nanoparticles with high biocompatibility and resistance to proteolytic digestion, macromolecules characterized by their symmetry and 3-D globular architecture, consisting of a central core, inner branches, and outer surface. Dendrimers have a well-defined shape, a highly monodisperse size, and a chemical homogeneity resulting from their repetitive branched pattern. In addition, they have significant advantages over linear polymers in that they have a higher loading capacity, a larger number of high-density surface functionalities that allow them to conjugate with other components. These nanocarriers called dendriplex, easily encapsulate siRNA and are optimal for delivery because their protonated amines induce an endosomal osmotic burst resulting in cytoplasmic accumulation of siRNA (Pishavar et al., 2021[118]; Subhan et al., 2021[142]; Yan et al., 2021[167]; Bholakant et al., 2020[16]).

### Cyclodextrin polymers

Cyclodextrins (CD) are crystalline, homogeneous, non-hygroscopic substances with different sizes, belonging to the family of tricyclic oligosaccharides composed of glucopyranose units, they are differentiated according to their number of units; αCD (6), βCD (7), and γCD (8), they have been used as excellent solubilizers and stabilizers thanks to their torus-like macro ring shape and their relatively hydrophobic cavity associated with an aqueous environment that allows them to form “host-guest” inclusion complexes, where the dissolved CD (host) allows energetically disadvantaged water molecules to move into their cavities with the “guest” molecule (ions, proteins, or oligonucleotides). Cyclodextrin polymers can be defined as molecules containing more than two covalently linked CD units, they are used to provide an alternative to conveniently deliver hydrophobic/hydrophilic molecules, thus, these systems are nanocarriers that could provide safe, effective, and targeted delivery of siRNA (Xu et al., 2021[162]; Pandey et al., 2022[113]; Pandey, 2021[112]; Mousazadeh et al., 2021[105]; Petitjean et al., 2021[117]; Liu et al., 2021[91]; Yao et al., 2019[168]; Ceborska, 2017[23]).

### Miscellaneous polymeric nanocarriers for siRNA delivery

Some polymeric nanocarriers are shown in Table 3(Tab. 3) (References in Table 3: Bai et al., 2020[11]; Cao et al., 2011[18]; Chen et al., 2014[27]; Craparo et al., 2020[31]; Fliervoet et al., 2020[39]; Ibaraki et al., 2020[60]; Kala et al., 2014[67]; Kaneshiro and Lu, 2009[68]; Lee et al., 2016[84]; Li et al., 2021[85]; Misra et al., 2014[101]; Noske et al., 2020[108]; Pan et al., 2019[111]; Salzano et al., 2015[129]; Sun et al., 2011[143]; Tang et al., 2015[146]; Wen et al., 2020[159]; Xiong and Lavasanifar, 2011[161]; Yuan et al., 2020[172]; Zheng et al., 2013[174]; Zhu et al., 2010[176], 2014[177]; Zou et al., 2020[180]), where the outstanding use of polymers such as PEG, PCL, PEI, and PNIPAM is observed. At the same time, it is observed that polymeric nanocarriers are conjugated with ligands, such as peptides, folic acid, and hyaluronic acid, and even hybrid polymeric nanocarriers composed of inorganic nanoparticles are observed. In general, these nanocarriers have sizes ranging from 7 to 591 nm, toxicity of less than 50 %, and gene knockdown ranging from 20 and 90 % *in vitro*, and are mainly used for melanoma, administered by IV injection. Additionally, other nanocarriers designed for the oral administration of siRNA, proposed by He et al. (2020[56]) are presented, these nanocarriers were composed of mannose-modified trimethyl chitosan-cysteine (MTC) and anionic cross linkers including TPP, HA, and Eudragit® S100, their properties were a size range between 120 and 225 nm and a ζ potential of 18-37 mV, they also showed an effective *in vitro* TNF-α knockdown of 25-75 % in Raw 264.7 cells and no significant toxicity (<10 %). These results in simulated gastric fluid are due to mucoadhesive properties of the three functional groups (trimethyl, thiol, and mannose) of the nanocarrier that promote oral absorption and the use of Eudragit® S100 that does not dissolve the system down to a specific pH.

## Nanocarriers Composed of Inorganic Nanoparticles

Inorganic nanoparticles (INPs) for siRNA delivery are generally composed of different types such as metallic nanoparticles, where gold nanoparticles stand out, super-magnetic nanoparticles, mainly iron oxides, semiconductor nanoparticles such as quantum dots, and ceramic nanoparticles, mainly mesoporous silica. INPs have emerged as valuable building blocks with continuous breakthroughs, particularly in their optical, electronic, magnetic, and catalytic properties, making them capable of detecting, diagnosing, and treating many diseases, and thus have numerous biomedical applications, including siRNA therapy. Moreover, INPs are synthesized through a variety of methods, creating extremely organized three-dimensional structures which can be modified with ligands to improve their affinity, they also have fairly attractive advantages such as precise control of nanocarrier size, high loading efficiency, control of API release, tunable surface properties, inertia, high stability, good reproducibility, easy cellular absorption, long useful life, and very attractive physical properties, making them prominent as theragnostic agents and recently as functional nanocarriers for siRNA and chemotherapeutic agents (Lins et al., 2021[89]; Torres-Vanegas et al., 2021[149]; Khan et al., 2021[71]; Khalid et al., 2020[70]; Khurana et al., 2019[72]).

For effective siRNA delivery, it is essential that these nanocarriers have external functionalization. Thus, INPs can form a coordination network between siRNA and organic nanoparticles, which generally increase their efficiency by improving their biocompatibility and protecting them from oxidation. In addition, INPs can be anchored to siRNA by physical adsorption, covalent coupling, or metal-ligand interactions. This versatility in incorporating siRNA has caused some of these nanocarriers to reach the advanced stage for clinical development, although most of them are still in the early stages (Zou et al., 2021[179]; Yau et al., 2021[169]; Jiang and Thayumanavan, 2020[65]; Charbe et al., 2020[24]).

### Metal-based nanoparticles: AuNPs

Among the nanocarriers composed of metallic nanoparticles, gold nanoparticles (AuNP) are commonly used since they have unique biochemical properties and can be created with a wide versatility of shape, size (∼15-50 nm using the Turkevich method), and tunable surface charges, they also have good properties such as non-toxicity, biocompatibility and can be easily adsorbed to the surface of APIs or can bind through covalent thiol bonds. In addition, these nanocarriers can induce a controlled release through different strategies and offer unique optical and electronic properties due to their strong localized surface plasmon resonance (LSPR). Gold nanoparticles coated with polymers or conjugated to another molecular compound have been extensively studied as siRNA delivery systems, since they have successfully demonstrated to be effective in gene knockdown, have no detectable off‐target effects, and also provide a photothermal therapeutic effect as a secondary function, making them even more attractive as nanocarriers (Pylaev et al., 2021[120]; Aghamiri et al., 2021[1]; Moore and Chow, 2021[102]; Gumala and Sutriyo, 2021[49]).

### Base-magnetic nanoparticles: SPIOs

Magnetic nanoparticles (MNPs) are a new type of magnetic nanocrystals composed of iron, nickel, cobalt, or magnesium. Iron oxides (Fe_3_O_4_ or Fe_2_O_3_) are the most important MNPs because they can produce strong paramagnetism, even superparamagnetism (SPIO, iron oxides with a diameter <50 nm) and are also safer than cobalt or nickel, which are reported to be more toxic. SPIOs possess advantages such as uniform size, large surface areas, high surface-to-volume ratio, a rapid transfection process and efficient biodegradability. In addition, SPIOs can provide target-oriented delivery because they interact with external magnetic fields (EMF) that allow them to be lead to target sites, even to hard-to-transfect and non-permissive cells, at the same time, they can provide molecular imaging and a magnetocaloric effect which can indirectly kill tumor cells, which is why these nanocarriers are used for siRNA delivery. These nanocarriers are highly efficient for releasing siRNA, thanks to magnetofection, a technique to enhance the efficiency of transfection of nucleic acid with EMF, but require improvements in their colloidal stability, so they usually have surface modifications using polymeric cross linkers that encapsulate these nanoparticles (Li et al., 2021[86]; Bassetto et al., 2021[14]; Maurer et al., 2021[98]; Liu et al., 2021[90]; Huang et al., 2021[59]; Dowaidar et al., 2017[35]).

### Semiconductor-based nanoparticles: QDs

Quantum dots (QDs) are colloidal semiconductor nanocrystals with sizes <10 nm, in general, their structure consists of a core and shell, composed of group II-VI elements (CdTe, ZnS, and CdSe), group III-IV elements (InAs and InP) or group III-IV elements IV-VI (CS, CSe, PbS, and PbSe). They can be classified into three main types: (1) according to their composition/structure: core-type (formed with a single component), core-shell type (core encapsulated by a semiconducting substance) and alloyed (formed with two semiconducting materials), (2) according to the material used for their preparation: semiconducting QDs and carbon/graphene QDs and (3) according to their size; large (5-6 nm) and small (2-3 nm). QDs have optical properties, absorbance and photoluminescence dependently allowing *real-time in situ *monitoring delivery of APIs. Additionally, they have long-term stability, wide-field excitation, an extensive emission spectrum and non-toxic effects, making them great candidates for theranostic therapy in conjunction with siRNA, however, these nanocarriers need superficial alterations employing hydrogel or covalent interlayer bonding to explicitly bind with nucleic acids (Singh et al., 2021[138]; Gidwani et al., 2021[47]; Tandale et al., 2021[145]; Khalid et al., 2020[70]; Kim et al., 2017[75]).

### Ceramic-based nanoparticles: MSNs

Ceramic nanoparticles are a relatively new type of porous inorganic nanoparticles for siRNA delivery, composed of silica, titanium oxide, calcium phosphate and alumina. These ceramic nanoparticles provide a tunable nanocarrier in both pore diameter (2-50 nm) and pore volume (> 0.9 cm^3^/g), as well as surface functionalization capability, high surface area, good biocompatibility, degradability, high loading capacity and chemical inertness. Mesoporous silica nanoparticles (MSNs) are the most relevant ceramic nanoparticles for siRNA delivery, having hundreds of empty channels that assemble into two- or three-dimensional porous structures, where they can load APIs such as siRNA. On the other hand, they are protonated by amination or coating with cationic polymers to enable electrostatic interactions with siRNA. Moreover, they can be functionalized with ''molecular gates” and have external stimuli to allow charge delivery to be triggered (Gao et al., 2021[42]; Yau et al., 2021[169]; García-Fernández et al., 2021[44]; Taleghani et al., 2021[144]; Lins et al., 2021[89]).

### Miscellaneous inorganic nanocarriers for siRNA delivery

To conclude this classification, Table 4(Tab. 4) (References in Table 4: Amani et al., 2021[6]; Cao et al., 2019[19]; Chen et al., 2009[25]; Kim et al., 2019[76]; Meng et al., 2010[100]; Qiao et al., 2021[121]; Shaabani et al., 2021[134]; Taschauer et al., 2020[147]; Wang et al., 2017[156]; Xue et al., 2021[165][166]) shows some examples of inorganic nanocarriers, where it is observed that Au-NPs (metallic nanoparticles) are the predominant INPs formulated, furthermore, all these nanocarriers are hybrid systems mainly with polymeric nanoparticles, also have properties such as size around 60-278 nm, toxicity less than 40 %, and gene knockdown in a range of 47-90 % *in vitro*. On the other hand, they are mainly used for breast cancer, administered by IV injection. Finally, nanocarriers designed for oral administration of siRNA are presented, proposed by Hosseini, et al. (2020[58]) which were capsules composed of freeze-dried calcium phosphate- polyethylene glycol nanoparticles (CaP-PEG) and trehalose nanoparticles with an outer layer of Eudragit® L100 (EL), chitosan (CS), cellulose acetate phthalate (CAP), hydroxypropyl methylcellulose (HPMC) or/and polyvinyl alcohol (PVA) as an enteric coating. These nanocarriers had a size range of 45 and 65 nm, PDI of 0.16-0.40, potential ζ of 16-18 mV, EGFP knockdown of 21-43 % *in vitro* in HeLa cells, and significant toxicity of around 50-20 % attributed to some polymers used as mucoadhesive excipients. 

## Supplementary Perspective for Nanocarrier Development

The success of siRNA-based therapeutics depends largely on their delivery system, thus requiring the use of nanocarriers that are at least: (i) biocompatible, biodegradable and non-immunogenic/non-toxic, (ii) non-sensitive to serum nucleases during transit through systemic circulation, (iii) specific for target cells while avoiding other tissues, and (iv) able to enter the cell membrane, the cellular environment and the endosomal pathway (Ge et al., 2021[45]; Tenchov et al., 2021[148]; Sharma et al., 2020[135]; Mahmoodi Chalbatani et al., 2019[95]).

The foregoing nanocarriers showed propitious characteristics conducive to siRNA delivery, in general, it was observed that almost all of them are designed for IV administration, regardless of the target. Although IV administration allows 100 % bioavailability of the API, it also had several limitations related to the invasiveness of the API administration process (pain at the injection site, patient discomfort, allergic reactions, scarring, etc.), this aspect should be considered especially for the treatment of chronic degenerative diseases such as cancer, which is one of the main approaches for the use of siRNA as a treatment and/or adjuvant, hence it is necessary that the development of nanocarriers also focuses on an oral administration of siRNA. In addition, this kind of administration can represent a potent modality for treating many gastrointestinal diseases such as inflammatory bowel disease (IBD), irritable bowel syndrome, and colon cancer, without adverse systemic effects (Tran and Park, 2021[150]; El‐Mayta et al., 2021[36]; Kanugo and Misra, 2020[69]). 

**Figure 3:**
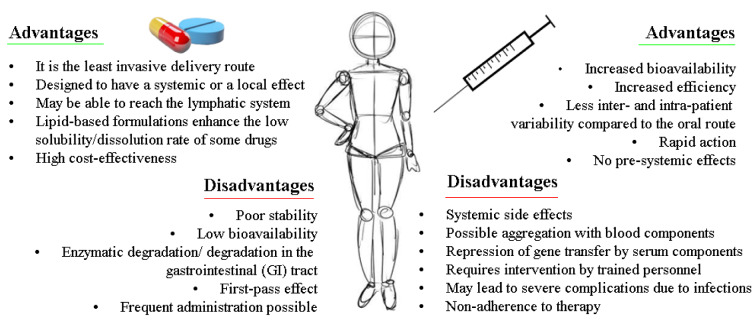
Advantage and disadvantage in oral and intravenous route for siRNA delivery, (Antimisiaris et al., 2021; Fumoto et al., 2021; van den Berg et al., 2021; Lorscheider et al., 2021; Hanna and Mayden, 2021)

It is well known that oral administration requires several efforts to deliver APIs, even more than parenteral administration (see Figure 3(Fig. 3); References in Figure 3: Antimisiaris et al., 2021[7]; Fumoto et al., 2021[40]; van den Berg et al., 2021[152]; Lorscheider et al., 2021[92]; Hanna and Mayden, 2021[52]), since it is a complex process that can be affected by different factors such as physiological and cellular barriers, in particular, it was shown in some studies that naked siRNA can withstand gastric challenges for one hour at physiological temperature, but is inevitably degraded by nucleases, thus siRNA necessarily needs a nanocarrier that can avoid enzymatic digestion, overcome GI mucus barriers, and facilitate their delivery into target cells (Rehman et al., 2021[125]; Ruiz-Picazo et al., 2021[127]). 

Some nanocarriers have been studied for oral siRNA administration; these nanocarriers are mainly composed of polymers and lipids. An example of oral administration was proposed by (Wang et al., 2021[158]), they formed a lipoplex with folic acid-conjugated ginger-derived lipid and siRNA. Although polymers are good absorption enhancers (~bioavailability) and have benefits such as controlled drug release, they cannot provide a satisfactory solution due to their associated toxicities, so lipid-based drug delivery systems (LBDDS) have been frequently proposed in recent years. These nanocarriers have advantages such as low toxicity, low cost, affordable scale-up manufacture, high biocompatibility, high drug loading efficiency and recruit a range of lipid digestion pathways in the GI tract that play a decisive role in the drug absorption process (Ashkar et al., 2022[9]; Plaza-Oliver et al., 2021[119]; Zu et al., 2021[182]; Tran et al., 2018[150]).

LBDDS can be classified into three types, previously mentioned two types: vesicular systems (micelles and liposomes) and solid lipid systems, the last type is an emulsion system, which is a novel approach for oral siRNA administration, especially the self-nanoemulsifying drug delivery system (SNEDDS). This system is composed of dissolved API, long and/or medium-chain triglyceride oils, high concentrations of non-ionic surfactants with HLB>12, and co-solvents to reduce interface between the oil and the aqueous medium, spontaneously forming fine oil-in-water nanoemulsions (o/w) *in situ* in the GI tract thanks to the stomach and small intestine motility (peristalsis) and the aqueous medium of the GI fluids, in a process called self-nanoemulsion (Dalal et al., 2021[33]; Xu, et al., 2021[164]; Morakul, 2020[103]; Sokkula and Gande, 2020[141]; Knaub et al., 2019[79]; Krstić et al., 2018[82]; Cherniakov et al., 2015[29]).

SNEDDS is an ideally isotropic and thermodynamically stable mixture, with droplet sizes below 200 nm thus having a large interfacial surface area for dispersion into the GI fluid, it is mainly designed to increase the solubility and permeability of APIs with lipophilic characteristics, however it has recently started to be used to improve the oral administration of hydrophilic macromolecules such as siRNA (nucleic acids), in such a way that the rate of drug dissolution, its absorption, digestion, and bioavailability can be improved. In addition, SNEDDS has a high drug loading capacity, is easy to manufacture and scale-up, it has good kinetic stability after dispersion in an aqueous medium, requiring a minimum amount of energy for dispersion and preparation, it has high physical stability during storage, decreases the first-pass effect and enhances penetration of highly lipophilic APIs into the intestinal membrane through the recruitment of intestinal lymphatic transport (Okonogi et al., 2021[110]; Jain et al., 2021[64]; Mehanna and Mneimneh 2020[99]; Buya et al., 2020[17]; Cardona et al., 2019[20]; Gilani et al., 2019[48]; Ng and Rogers, 2019[106]; Rehman et al., 2017[126]).

The main strategy for incorporating nucleic acids into SNEDDS includes reducing their hydrophilicity by pairing hydrophobic ions, this method is based on replacing the negatively charged counterions with positively charged surfactants or cationic lipids. The first work on this was presented by (Hauptstein et al., 2015[54]), where pDNA complexes were formed using 5 different cationic components highlighting the use of cetrimide. These complexes were properly dissolved in SNEDDS thus the pDNA was successfully incorporated, obtaining a nanocarrier with an effect against enzymatic degradation and a good transfection efficiency of HEK-293 cells. Furthermore, Mahmood et al. (2016[94]) presented a similar work based on pDNA-cetrimide, where the transfection efficiency of SNEDDS was improved by the incorporation of a cell-penetrating peptide (TAT-OL). Finally, the most recent work, to our knowledge, was presented by Kubackova et al. (2021[83]) where SEDDS loaded with oligonucleotide (OND)-DDAB or DOTAP complexes were prepared and characterized using the hydrophobic ion pairing technique. This nanocarrier was a viable delivery system across the Caco-2 monolayer and was protected OND in the GI tract.

## Conclusions

The use of siRNA as a mediator of gene silencing is a novel alternative for the treatment of various diseases, its advantages over traditional RNA delivery make it a suitable tool for the improvement of the bioavailability of a therapeutic effect. As a result, a wide range of nanocarriers for the transport and delivery of siRNA has been developed, however, only a few of them are in clinical trials.

The classification of nanocarriers outlined in this review is a suggestion, which considers the nature (organic and inorganic) of single ingredients, their chemical structure (lipids and polymers), and the shape of the nanocarrier (liposomes/polymersomes and micelles). However, almost all nanocarriers are hybrid systems and should not be limited to a single classification; a relevant example of this are inorganic nanocarriers, which are generally composed of siRNA complexes with organic nanoparticles.

These nanocarriers have proven to be stable, biocompatible, and effective *in vitro*, but only very few are designed for oral administration of siRNA. This approach has emerged to offer enhanced nanocarriers that can satisfy different needs, such as a targeted treatment for gastrointestinal diseases and nanocarriers that may facilitate adherence to treatments and do not affect the patients' quality of life. Therefore, there is a need to further explore the development of nanocarriers to obtain safe, biocompatible, and suitable biopharmaceutical tools that allow the enhancement of the absorption and targeting of siRNA for effective therapeutic alternatives.

## Declaration

### Declaration of competing interests

The authors declare no conflict of interest.

### Acknowledgments

The authors would like to acknowledge the financial support received from the National Council for Science and Technology (CONACyT, Mexico) through the grant: CONACyT-CF 2019-263379. This work was carried out as part of the activities of the National Laboratory for the Research and Development of Radiopharmaceuticals (LANIDER-CONACyT; Mexico). The authors also appreciate the graduate student scholarship granted to Aideé Morales-Becerril through the National Quality Postgraduate Program (PNPC; CONACyT, Mexico). She is a graduate student from the M.D. program in Science and Pharmaceutical Technology at UAEMex.

## Figures and Tables

**Table 1:**
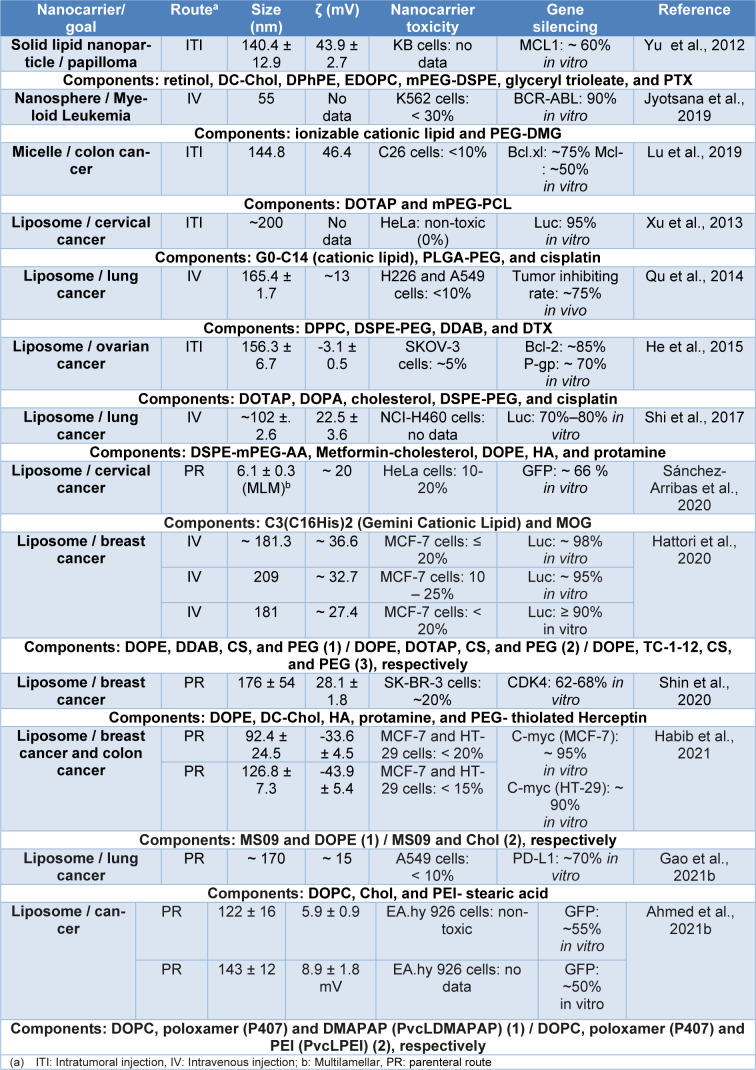
Nanocarriers composed of lipid nanoparticles

**Table 2:**
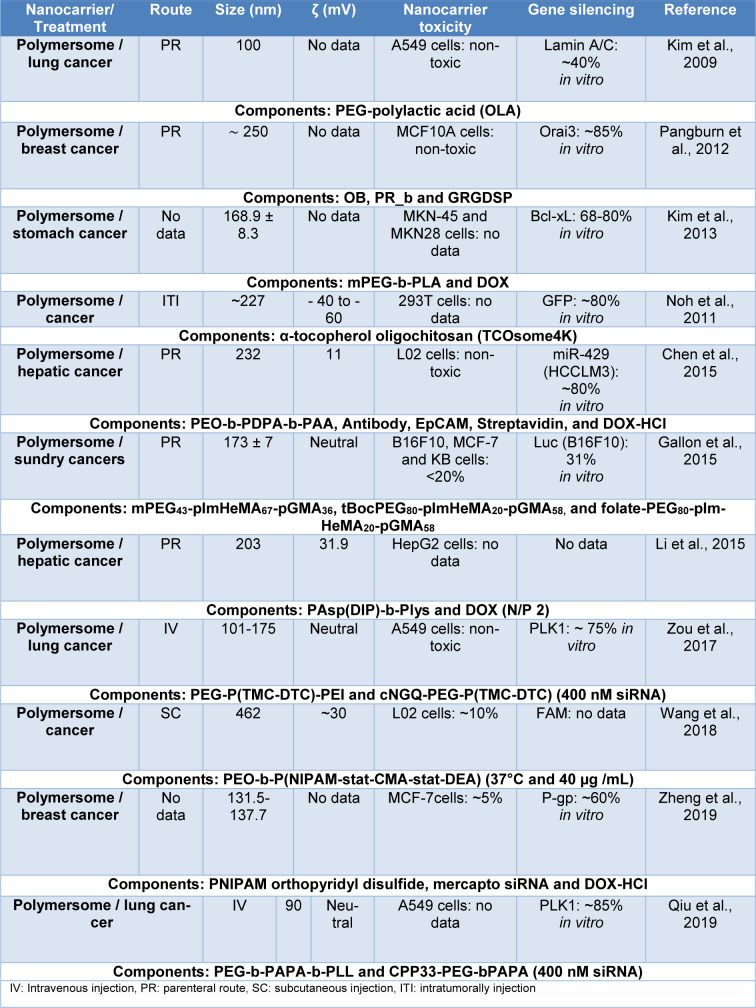
Nanocarriers composed of polymeric nanoparticles

**Table 3:**
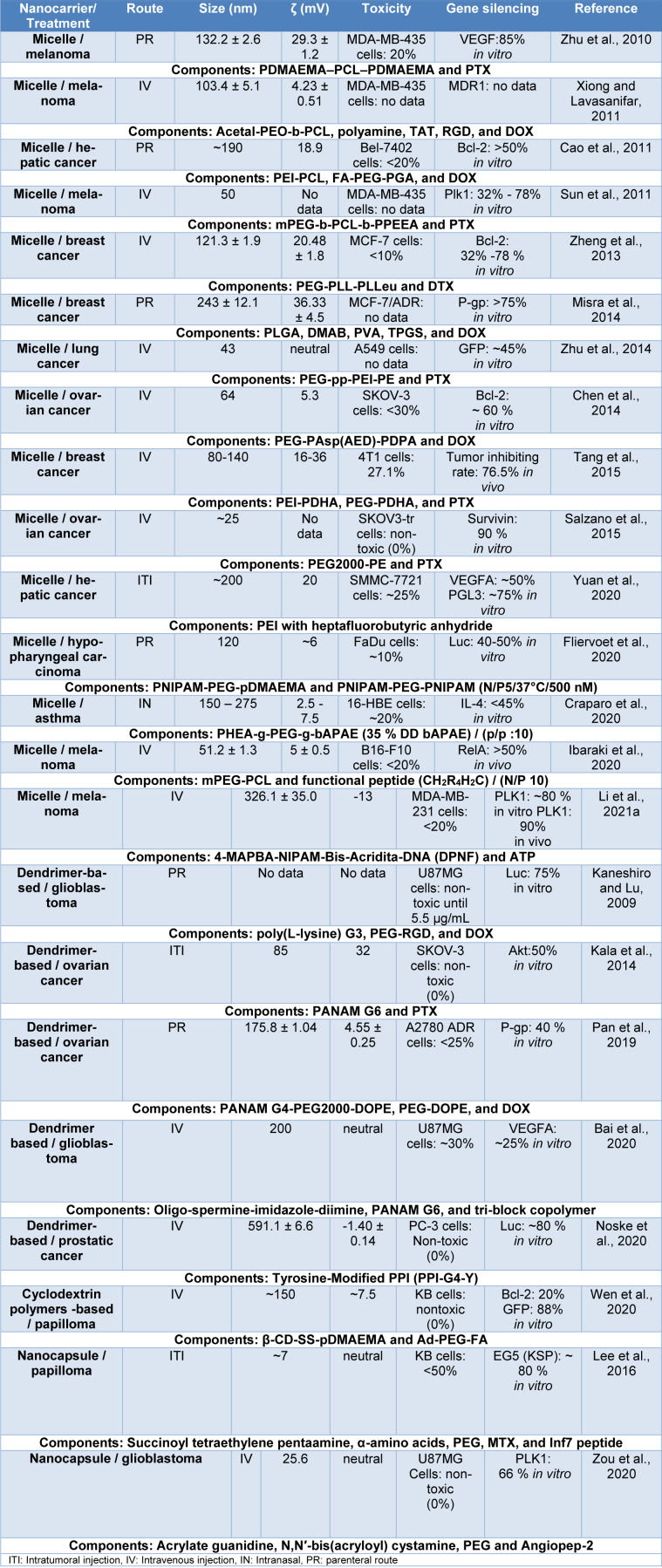
Nanocarriers composed of miscellaneous polymeric nanoparticles

**Table 4:**
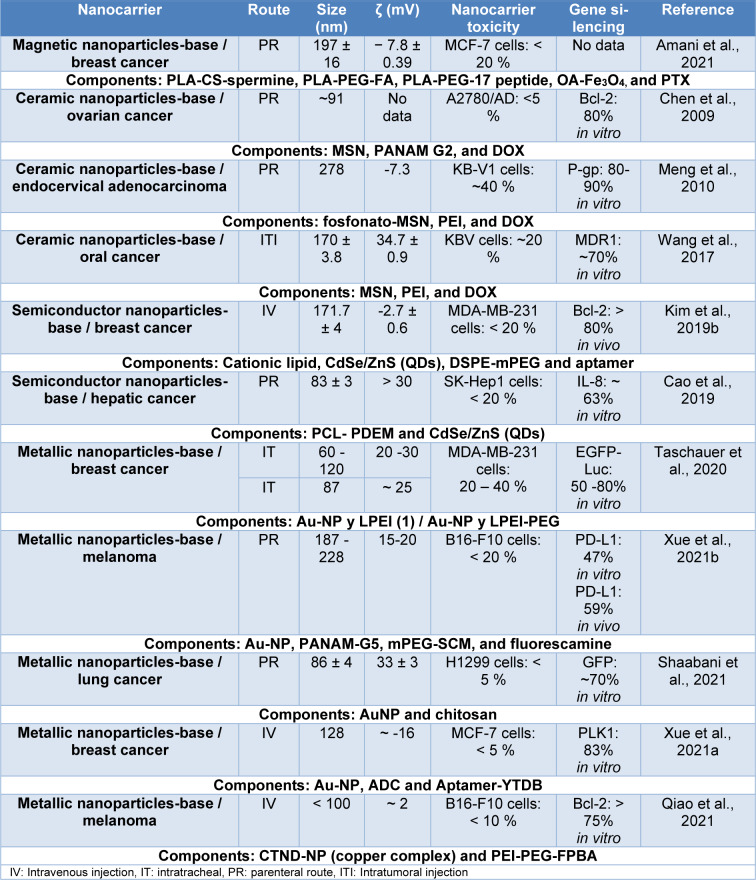
Nanocarriers composed of inorganic nanoparticles
